# VAV2: a novel prognostic marker and a druggable target for adrenocortical carcinoma

**DOI:** 10.18632/oncotarget.21448

**Published:** 2017-10-02

**Authors:** Carmen Ruggiero, Enzo Lalli

**Affiliations:** Enzo Lalli: CNRS, NEOGENEX CNRS International Associated Laboratory, Institut de Pharmacologie Moléculaire et Cellulaire, Université Côte d’Azur, Sophia Antipolis, Valbonne, France

**Keywords:** cancer, adrenal cortex, guanine nucleotide exchange factor, cytoskeleton, cell invasion

Adrenocortical carcinoma (ACC) is a rare endocrine malignancy with a dismal prognosis. Current polychemotherapeutic cytotoxic regimens or targeted therapies have a limited efficacy in the advanced metastatic stage of the disease. The molecular mechanisms underlying adrenocortical tumorigenesis remain to be fully elucidated.

An important player involved in ACC pathogenesis is the transcription factor steroidogenic factor-1 (SF-1), which has a pivotal role in the development of adrenal glands and gonads and in the control of steroidogenesis [1]. Increased SF-1 dosage is associated with enhanced adrenocortical tumor cell proliferation, development of adrenocortical tumors in mice and confers a more severe prognosis to adult adrenocortical cancers [2, 3].

We analyzed the mechanisms by which SF-1 controls gene expression in ACC cells and showed that SF-1 regulates multiple gene categories according to its dosage [2]. SF-1 dosage-dependent target gene products are implicated in a variety of cellular processes and control pivotal signalling pathways [2].

**Figure 1:**
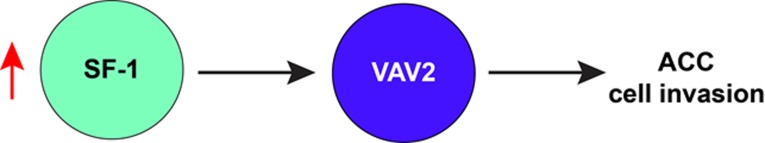
VAV2 is upregulated by increased SF-1 dosage In ACC, an increased SF-1 dosage transcriptionally upregulates expression of the VAV2 GEF. Increased VAV2 levels drive cancer cell invasion and are associated with shorter survival in ACC patients.

In our recent study “Dosage-dependent transcriptional regulation of VAV2 by Steroidogenic Factor-1 drives tumor cell invasion” [4], we focused on one of the SF-1 dosage-dependent target genes identified in our previous genomic studies, VAV2. It encodes a guanine nucleotide exchange factor (GEF) for Rho small GTPases, which has an important function in many aspects of tumor biology [5]. Our findings revealed a transcriptional regulatory loop involving upregulation of VAV2, driven by overexpression of SF-1 in ACC (Figure 1). VAV2 is an essential factor driving the invasive phenotype of ACC cells, whose expression is robustly correlated with prognosis in ACC patients and represents a potentially druggable target [4].

Our findings put in direct relationship dosage-dependent transcriptional regulation by SF-1 with VAV2, providing the first example of direct transcriptional regulation of a GEF by an oncogenic transcription factor [4]. Classically, GEF activation in cancer rather involves activation by upstream tyrosine kinases or overexpression due to genomic imbalances. Indeed, the known mechanism of VAV2 activation in cancer consists in the phosphorylation of regulatory tyrosine residues by oncogenic kinases [5]. VAV2 is hyperactivated in head and neck squamous cell carcinoma through an epidermal growth factor (EGFR)-dependent autocrine loop [5]. In turn, it is responsible for slowing down receptor internalization and degradation, further increasing EGF signalling. In addition to modulate Rho small GTPase activity and cytoskeleton remodeling, VAV2 and VAV3 control a transcriptional program that regulates specific steps of metastatic dissemination of breast cancer cells to the lungs [5]. Moreover, Vav2^−/−^Vav3^−/−^ double knockout mice display reduced xenograft tumor growth when transplanted with lung or melanoma cells. This suggests a role for VAV proteins in the host microenvironment, highlighting the multiple ways by which those GEFs participate to tumorigenesis.

Elevation of Rho small GTPase GEF expression and/or activity is a common phenomenon during cancer progression [6]. Thus, Rho-GEFs represent attractive therapeutic targets for cancer [6]. Targeting the binding of Rho small GTPases to GEFs is a rational strategy to inhibit Rac activity [6]. Because of their important role in tumor spreading and metastasis, VAV proteins have been proposed to be potentially druggable by small molecules and amenable to development of novel tools for cancer therapy. The recent development of inhibitors of RAC1-VAV2 association [7] has provided the proof of principle that this interaction represents a druggable target in cancer. The extensive analysis of the mechanism of action of Brefeldin A, the first known GEF inhibitor, has led to the general concept of « interfacial inhibition » [6]. It refers to inhibitors, like the recently developed Vav2-Rac interaction inhibitor Ehop-016 [7], that act by stabilizing protein complexes and targeting regions in or near interfaces. Our findings open the way to the development of VAV2 inhibitors as novel targeted pharmacological tools against advanced ACC, which is of seminal importance as the lack of effective therapeutic options makes the need for more selective and specific treatments urgent.

Increased SF-1 abundance is significantly correlated to shorter progression-free survival (PFS) and overall survival (OS) of ACC patients [3]. We showed a strong correlation between SF-1 and VAV2 expression in tumors [4]. Those clinical data together with the results obtained in our ACC cellular model strongly suggest that SF-1 is a major element responsible for higher VAV2 expression in ACC cases with a more severe prognosis. A remarkable clinically relevant outcome of our study consists in the highly significant correlation between high VAV2 abundance and reduced ACC patient survival [4]. Those findings are particularly robust as they derive form three different cohorts of patients using different platforms to assess both VAV2 mRNA expression and VAV2 protein abundance [4]. Moreover, in our series of cases, we appreciated a significant correlation between VAV2 H-score and the Ki67 labeling index (LI) [4], which has a major prognostic role in localized ACC after complete resection. Those data prompted us to carry out a large study involving ACC cases provided by multiple European institutions to assess the prognostic value of VAV2 expression in ACC and to compare and integrate it to the Ki67 LI. This study shows that the tumoral VAV2 H-score is significantly correlated to PFS and OS in ACC patients [8]. The combined assessment of VAV2 expression and Ki67 LI improves prognostic prediction in ACC [8].

By a “bottom-up” strategy, starting from a cellular model and using the data collected from its genomic characterization, we thus identified VAV2 as a novel molecular player implicated in ACC malignancy. VAV2 is a potentially druggable target and a new prognostic marker suitable for clinical application.
